# Probable human origin of the SARS-CoV-2 polybasic furin cleavage motif

**DOI:** 10.1186/s12863-023-01169-8

**Published:** 2023-11-21

**Authors:** Antonio R. Romeu

**Affiliations:** Biochemistry and Molecular Biology, University Rovira i Virgili, Tarragona, Spain

**Keywords:** SARS-CoV-2, Polybasic furin cleavage motif, Human mRNA transcripts, Spike glycoprotein insertions, Bioinformatics

## Abstract

**Background:**

The key evolutionary step leading to the pandemic virus was the acquisition of the PRRA furin cleavage motif at the spike glycoprotein S1/S2 junction by a progenitor of SARS-CoV-2. Two of its features draw attention: (*i*) it is absent in other known lineage B beta-coronaviruses, including the newly discovered coronaviruses in bats from Laos and Vietnam, which are the closest known relatives of the covid virus; and, (*ii*) it introduced the pair of arginine codons (CGG-CGG), whose usage is extremely rare in coronaviruses. With an occurrence rate of only 3%, the arginine CGG codon is considered a minority in SARS CoV-2. On the other hand, Laos and Vietnam bat coronaviruses contain receptor-binding domains that are almost identical to that of SARS-CoV-2 and can therefore infect human cells despite the absence of the furin cleavage motif.

**Results:**

Based on these data, the aim of this work is to provide a detailed sequence analysis between the SARS-CoV-2 S gene insert encoding PRRA and the human mRNA transcripts. The result showed a 100% match to several mRNA transcripts. The set of human genes whose mRNAs match this S gene insert are ubiquitous and highly expressed, e.g., the ATPase F1 (ATP5F1) and the ubiquitin specific peptidase 21 (USP21) genes; or specific genes of target organs or tissues of the SARS-CoV-2 infection (e.g., MEMO1, SALL3, TRIM17, CWC15, CCDC187, FAM71E2, GAB4, PRDM13). Results suggest that a recombination between the genome of a SARS-CoV-2 progenitor and human mRNA transcripts could be the origin of the S gene 12-nucleotide insert encoding the S protein PRRA motif.

**Conclusions:**

The hypothesis of probable human origin of the SARS-CoV-2 polybasic furin cleavage motif is supported by: (*i*) the nature of human genes whose mRNA sequence 100% match the S gene insert; (*ii*) the synonymous base substitution in the arginine codons (CGG-CGG); and (*iii*) further spike glycoprotein PRRA-like insertions suggesting that the acquisition of PRRA may not have been a single recombination event.

**Supplementary Information:**

The online version contains supplementary material available at 10.1186/s12863-023-01169-8.

## Background

The key evolutionary step leading to the pandemic virus was the acquisition of the furin polybasic motif at the spike glycoprotein S1/S2 junction by a progenitor of SARS-CoV-2. In the first SARS-CoV-2 clinical isolates it was proline (P), arginine (R), arginine and alanine (A) (PRRA) [1–5]. Two of its features draw attention: (*i*) PRRA is absent in other known sarbecoviruses (lineage B beta-coronaviruses), including the newly discovered coronaviruses in bats from Laos and Vietnam, which are the closest known relatives of the covid virus [6, 7]; and, (*ii*) it introduced the pair of arginine codons (CGG-CGG), whose usage is extremely rare in coronaviruses [8, 9]. With an occurrence rate of only 3%, the arginine CGG codon is considered a minority in SARS CoV-2 [10, 11]. On the other hand, Laos and Vietnam bat coronaviruses contain receptor-binding domains that are almost identical to that of SARS-CoV-2 and can therefore infect human cells despite the absence of the furin cleavage motif [6, 7]. Based on these data, the aim of this work is to provide a detailed sequence analysis between the SARS-CoV-2 S gene insert encoding PRRA and the human mRNA transcripts [12, 13]. The result showed a 100% match to several mRNA transcripts suggesting the hypothesis of a probable human origin of the PRRA coding sequence acquired through recombination by a progenitor of the SARS-CoV-2.

## Results and discussion

### SARS-CoV-2 S gene 12-nucleotide insert and human mRNA transcripts

**Fig. 1:**
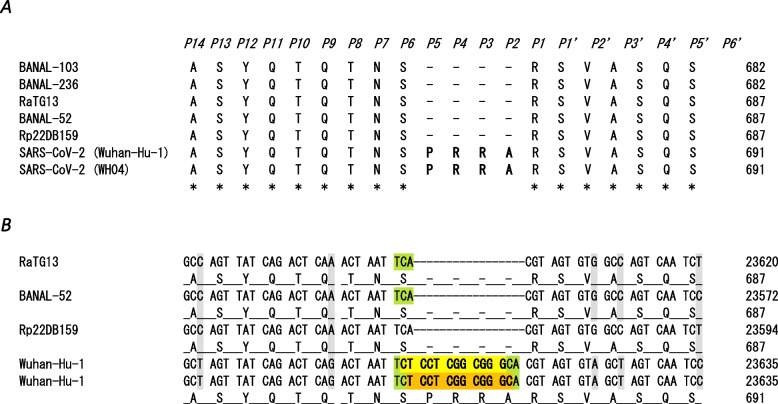
SARS-CoV-2 spike glycoprotein polybasic furin cleavage site. Fragment of a multiple sequence alignment covering the SARS-CoV-2 spike glycoprotein polybasic furin cleavage site. The first line at the top indicates the positions, in a P14-P6’ nomenclature, of the canonical structure of a furin site in a given protein. The specific cleavage site is between positions P1 and P1’. The core regions is between positions P6–P2’ and there are two flanking solvent accessible regions: P7–P14 and P3’–P6’ [15]. Part **A**. Fragment of the protein multiple sequence aligment including Laos bat *Rhinolophus* coronaviruses BANAL-52 (GISAID, EPI_ISL_4302644: 21512–25,321), BANAL-103 (GISAID, EPI_ISL_4302645: 21498–25,294), BANAL-236 (GISAID, EPI_ISL_4302647: 21538–25,344), Vietnam bat *Rhinolophus pusillus* Rp22DB159 coronavirus (GenBank: WLJ60537.1 coded by OR233302.1:21533..25342 genome), Bat coronavirus RaTG13 (GenBank: QHR63300.2 coded by MN996532.2: 21560..25369 genome) and the reference SARS-CoV-2 sequences (isolates Wuhan-Hu-1 and WH04) [14]. The SARS-CoV-2 polybasic insert (PRRA) is denoted in bold. Strictly conserved amino acids are denoted by *. The amino acid position is indicated at the numbers on the right. Part **B**. Fragment of the codon alignment. For simplicity, from the Laos coronavirus only the BANAL-52 sequence has been included. The two possibles 12 nucleotide fragment encoding PRRA inserted within the S680 codon are highlighted in yellow and orange, respectively. The S680 TCA codon is denoted in green. The differences at the third codon position are denoted in gray. The protein and genome sequence position is indicated at the numbers on the right

With coordinate based on SARS-CoV-2 reference sequence [14], the S gene 12-nucleotide fragment encoding the PRRA polybasic motif is located within the TCA S680 codon. Figure 1 shows the two possibilities: “CT CCT CGG CGG G” or “T CCT CGG CGG GC” depending on whether the insertion took place between positions 1 and 2 or 2 and 3 of the TCA codon, respectively. Here the reverse complement of the two possible S gene inserts have been included in the similarity analysis with the mRNA transcripts. In the creation of new SARS-CoV-2 particles in infected human cells, both ssRNA(+) and ssRNA(−) SARS-CoV-2 genomes that coexist through the viral RNA-dependent RNA polymerase (RdRp) [16] are equally important with respect to a possible recombination with human mRNAs.

Sequence analysis showed that the SRAS-CoV-2 S gene 12-nucleotide fragments, potentially involved in the PRRA coding, 100% match to several NCBI human mRNA RefSeq transcripts (Tables 1, 2, 3, and 4).

**Table 1:**
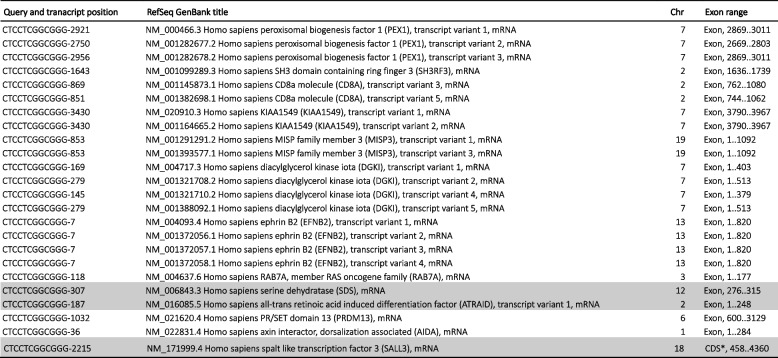
Human NCBI NM_RefSeq Transcripts (curated protein coding) that match the CTCCTCGGCGGG SARS-CoV-2 furin cleavage insert

The rows highlighted in gray denote the transcripts whose genes also match TCCTCGGCGGGC (Table 2)

*: in gene annotation, coding sequences (CDS)

**Table 2:**
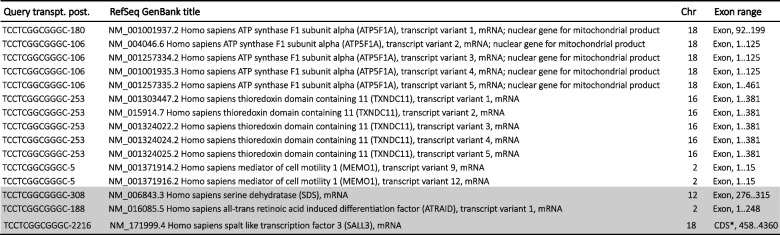
Human NCBI NM_RefSeq Transcripts (curated protein coding) that match the TCCTCGGCGGGC SARS-CoV-2 furin cleavage insert

The rows highlighted in gray denote the transcripts whose genes also match CTCCTCGGCGGG (Table 1)

*: in gene annotation, coding sequences (CDS)

**Table 3:**
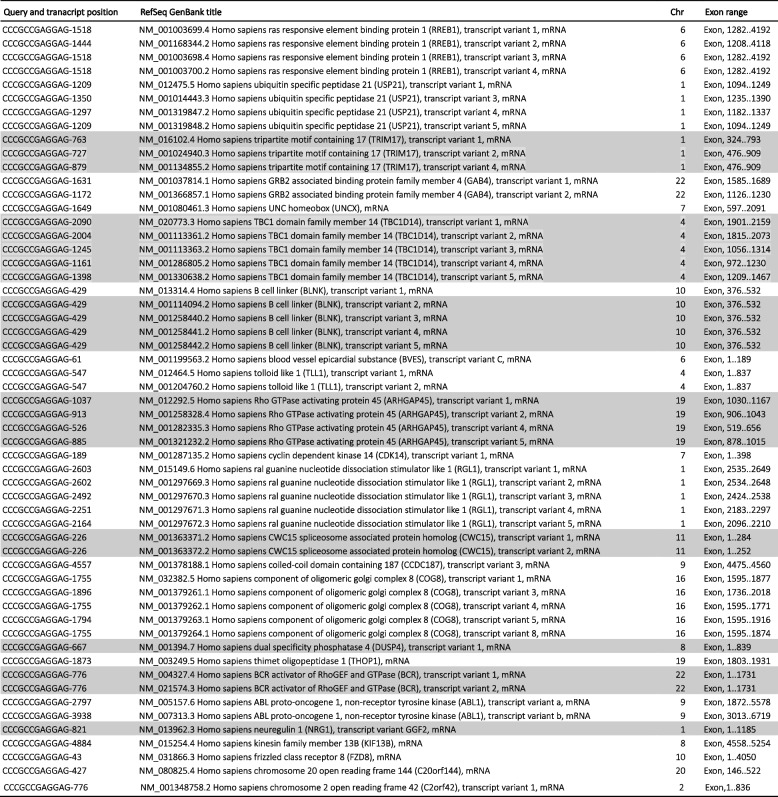
Human NCBI NM_RefSeq Transcripts (curated protein coding) that match the CCCGCCGAGGAG (CTCCTCGGCGGG reverse complement) SARS-CoV-2 furin cleavage insert

The rows highlighted in gray denote the transcripts whose genes also match GCCCGCCGAGGA (Table 4)

**Table 4:**
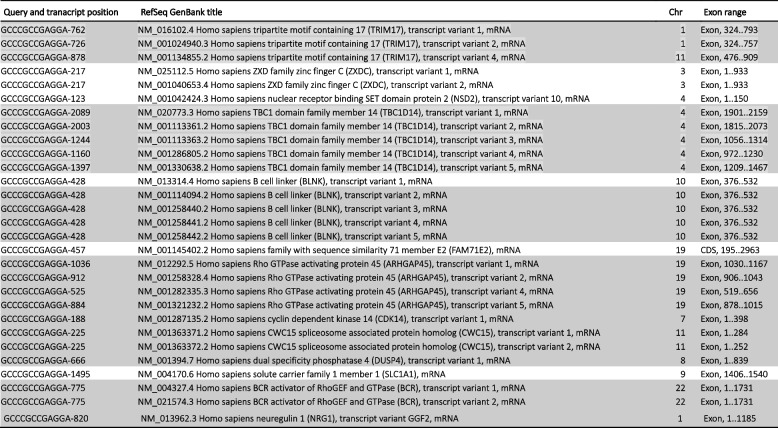
Human NCBI NM_RefSeq Transcripts (curated protein coding) that match the GCCCGCCGAGGA (TCCTCGGCGGGC reverse complement) SARS-CoV-2 furin cleavage insert

The rows highlighted in gray denote the transcripts whose genes also match CCCGCCGAGGAG (Table 3)

Tables 1, 2, 3, and 4 show the curate RefSeq mRNA protein-coding human transcripts, labelled “NM_” that matched the S gene insert. They group together 44 human genes and their variants. All of the human mRNA fragments matching the 12-nucleotide viral sequences are located in specific exons. No functional trend has been observed in the gene products. Also, these genes are distributed throughout all chromosomes. This suggests that these human transcripts could be good candidates as donors of mRNA template in a potential recombination link to a SARS-CoV-2 furin cleavage motif. However, the presence of the CGG-CGG in these human mRNA transcripts does not necessarily imply there would be an arginine pair in the gene product. It may not be in the reading frame.

Tables 1S-4S show the results extended to the four series of human mRNA RefSeq transcripts: NM_ (curated mRNA protein-coding), NR_ (RNA non-protein-coding), XM_ (predicted model protein-coding) and XR_ (RNA predicted model non-protein-coding).

### Tissue-specificity of genes whose mRNA transcripts 100% match viral sequences

**Fig. 2:**
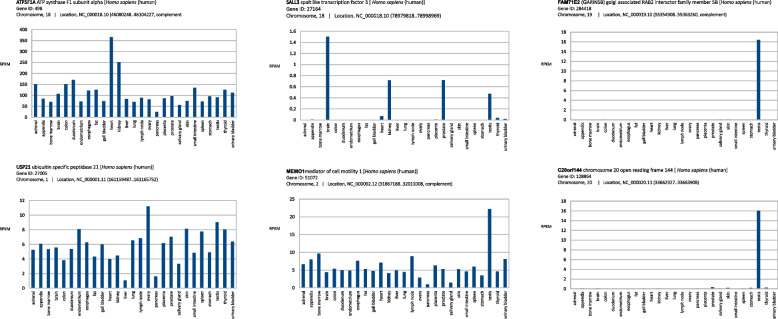
Tissue specificity of human genes matching SARS-CoV-2 S gene coding PRRA insert. Examples of tissue specificity of human genes (shown in Tables 1, 2, 3, and 4) whose transcripts match 100% with SARS-CoV-2 S gene insert encoded the PRRA polybasic furin motif. Expression pattern based on the Human Protein Atlas RNA-seq normal tissues NCBI BioProject Accession: PRJEB4337 ID: 231263. Data were download from NCBI Human Genome Resources, assembly GRCh38.p14. Units of transcript expression are normalized reads per kilobase of transcript, per million mapped reads (RPKM)

The set of human genes whose mRNAs match the SARS-CoV-2 S gene PRRA coding region (Tables 1, 2, 3, and 4) can be grouped into two categories in terms of tissue-specificity: (*i*) ubiquitous and highly expressed; and (*ii*) specific to target organs or tissues of the SARS-CoV-2 infection. As an example, Fig. 2 shows some examples of tissue specificity. In the first group, the alpha subunit of the ATPase F1 (ATP5F1) gene encodes the catalytic core of the mitochondrial ATP synthase; and the ubiquitin specific peptidase 21 (USP21) gene-encoding protein cleaves ubiquitin for recycling in intracellular protein degradation. In the second group there are genes with a tissue specificity in the brain, kidney, prostate and testis, which are targets in virus infection [17] (e.g., MEMO1, SALL3, TRIM17, CWC15). However, some genes must be highlighted as unique and highly expressed in the testis (e.g., CCDC187, FAM71E2, GAB4, PRDM13).

### Hypothesis of probable human origin of the SARS-CoV-2 polybasic furin cleavage site

The 100% match between the S gene insert encoding the furin polybasic motif and some human mRNA transcripts suggests that a recombination between the viral and human RNA could be the origin of that S gene insert. This is the proposal of the hypothesis on the probable human origin of the SARS-CoV-2 polybasic furin cleavage mptif, which agrees with a possibility already put forward by R. F. Garry and co-workers [1] that a progenitor of SARS-CoV-2 passed to humans, acquiring the PRRA during undetected human-to-human transmission.

Recombination is the common method by which viruses acquire new skills [18]. In this case the skill was the ability to interact with the human furin serin protease, which further aids the entry of SARS-CoV-2 into human cells.

### Evidence supporting the hypothesis

#### Evidence 1. Synonymous base substitution at the SARS-CoV-2 S gene arginine codons CGG-CGG

The arginine pair of the furin polybasic motif is essential for the SARS-CoV-2. It is evolutionarily strictly conserved. Although mutations of the variants also extend to the PRRA, the RR pair remains e.g., Delta P/R (RRRA), Omicron P/histidine, H (HRRA). In contrast, this key arginine pair is encoded by the “extremely rare” CGG-CGG codons in coronaviruses. So, everything suggests an evolutionary pressure in that point of the SARS-CoV-2 S gene.

Sequence analysis shows that a synonymous base substitution in the furin arginine pair code actually occurs. Based on the NCBI Virus database, a large sample of spike glycoprotein squences (with release date from January 1, 2020 to September 30, 2023) 8459 out of 3,494,735 (0.2420%) protein sequences showed arginine codon usage bias in one of the CGG-CGG codons (Table 5S). Also, based on data from the GISAID database, 155 out of 78,085 (0.1985%) showed the same arginine codon usage bias (Table 6S). The sequences that have a synonymous base substitution in one of the CGG arginine codons at the furin arginine pair cover several SARS-CoV-2 lineages and geographic regions.

When the analyses focused on specific SARS-CoV-2 lineages, results were significant. Based on a GISAID sample of isolates from the XBB.1.16.20 lineage 75 out of 354 (21.19%) showed a synonymous base substitution in the second arginine codon of the furin polybasic motif, the CGG-CGG code has mutated to CGG-CGT. Isolates from the EE.2 lineage, 533 out of 1021 (52.20%) the CGG-CGG was replaced by CGA-CGG. Furthermore, isolates from the CQ.2, CQ.1 and CQ.1.1 lineages the 100% of the analysed spike glycoprotein sequences showed arginine codon usage bias in one of the CGG pair. The results were as follows: CQ.2 lineage, 286 out of 287 (99.65%); CQ.1516 out of 516 (100%); CQ.1.1117 out of 177 (100%). Table 7S shows basic information of these SARS-CoV-2 lineage isolates.

These results suggest a SARS-CoV-2 S gene trend towards an arginine codon usage bias encoding the spike glycoprotein furin polybasic motif.

#### Evidence 2. PRRA-like insertions in the SARS-CoV-2 spike glycoprotein sequence

**Fig. 3:**
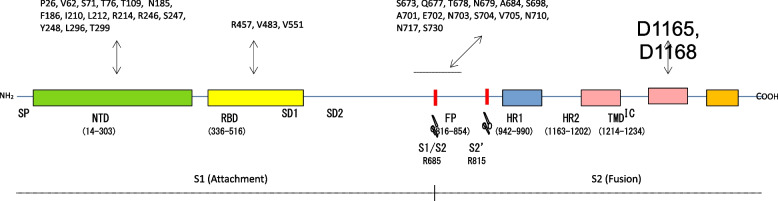
PRRA-like insertion along the SARS-CoV-2 spike glycoprotein. PRRA-like insertion positions in the SARS-CoV-2 spike glycoprotein. With coordinate based on SARS-CoV reference sequence [14], boxes on the top show the sequence positions of the PRRA-like insertions. The SARS-CoV-2 spike glycoprotein structural domains and cleavage sites are indicated. Protein length: 1273. At the bottom the S1 subunit and S2 subunit in each spike glycoprotein monomer are indicated. The coordinates of some of the domains are in parentheses. Acronyms: S1, subunit 1; S2, subunit 2; SP, signal peptide; NTD. N-terminal domain; RBD, receptor binding domain; SD1, subdomain 1; SD2, subdomain 2; FP, fusion peptide; HR1, heptad repeat 1; HR2, heptad repeat 2; TM, transmembrane region; IC, inracellular domain. Cleavage sites: S1/S2, R685/S686, by host (human) furin, paired basic amino acid cleaving enzyme (FURIN); S2’, R815/S816, by host transmembrane serine protease 2 (TMPRSS2) orcathepsin L (CTSL). The figure has been created based on data from [19–21]

Once SARS-CoV-2 emerged, the question is whether there have been further spike glycoprotein insertions similar to that of the furin polybasic motif in a SARS-CoV-2 progenitor. Based on the NCBI Virus database, the analysis of 2,315,308 spike glycoprotein sequences with no ambiguous characters showed many other PRRA-like insertions throughout the sequence, in the different spike glycoprotein structural domains (Fig. 3). Table 5 shows the inserted fragment, its position and the identification of the involved sequence. These insertions are PRRA-like because they satisfy the following requirements: (*i*) the S gene insert encoding a given S protein insert has to 100% match to human mRNA transcripts; and (*ii*) the related genes have to be ubiquitous and highly expressed genes or specific genes of target organs or tissues of virus infection.

**Table 5:** Spike glycoprotein PRRA-like insertions from SARS-CoV-2 isolates

Position	Insert	Genbank Accession	Number
		N-terminal domain	
P26	IEKN	UFA95489	1
V62	TSGNN	QZQ09947	1
S71	HLKD	UEP74721	1
T76	AVMSL	UEV78566	1
T109	ILWW	UHT93004	1
N185	MQAVS	UWL01218	1
	SARW	UYR39941	1
F186	TPAGG	WCF88733	1
I210	KKGA	UWJ32324	1
	RHAVLSG	WDD77904	1
L212	FMAE	UPH02236, UPH02715, UPL60987, UPL79201, UPL83322, UPL97779, UPS43182, UPW23099, UPW23870, UZZ69309, URP81062, URP81074, URP81794, URG88035, UQE96806, UQE28102	16
	TVGG	UPQ93706, UPQ95992, UYL90137, UYL90173, UPN04511, UQR90517, UQR92623, UQS02680, UQS06834, UQS05613, UQE37288	11
	NLTI	WED35345	1
	REPEDR	UJS73581	1
R214	ASPN	UIS34449	1
	DQAF	UAB38160	1
	EPEDN	UIG18418	1
R246	SEIE	QTQ48337	1
	TLRA	WCL14692, WBE99745, WBG79084	3
S247	LRAG	WBG79084, WCC34642, WBY86968, WBT65812	4
	SKWL	WCP01579, WDG34661, OQ439587, OQ673691, OQ445194, OQ458267, OQ193613	7
	SRWM	ZK92183, WAD73656, WAD78937, WAD79819,…. (see Table S1)	331
	SVGS	WCZ87602, OQ431694	1
	YGHT	ULC27716	1
	YHSD	UXM33786	1
	YRSCCIQ	ULP78342	1
Y248	AGTG	UQW64543	1
	HSDR	UXV06186	1
	KWLD	WGI99683	1
	RWMD	WGL43195	1
L296	HGHTF	UHG56576	1
T299	AVPY	UHJ33999	1
		Receptor binding domain	
R457	HYKYF	QVM41426	1
V483	EVQF	UKF78016	1
V551	EIPTS	UEZ97007, UFA03190, UEF49231	3
		Furin cleavage site (S1/S2 junction)	
S673	YSLS	QVL88657, QVL88693	2
T678	TQRA	WCJ55381	1
N679	GIAL	QSX93802	1
	KAVR	QTC70411	1
		S1/S2 region	
S698	LHHV	UVT41921	1
A701	GTNA	UPA68498	1
E702	CGPKKST	UNH71219	1
	LSSTE	UUH48256	1
	SLSSTA	UTV73082	1
N703	YSLSS	UWQ86862	1
S704	WCWL	URN60119	1
V705	GNICYT	UXB43176	1
N710	KPCNGVAG	UTH52964	1
N717	SHVV	UJT69381	1
S730	TNVS	UNW19168	1
		Heptad repeat 2	
D1165	WLSR	URF73848	1
D1168	ISGIDLGD	UHY88495	1

**Fig. 4:**
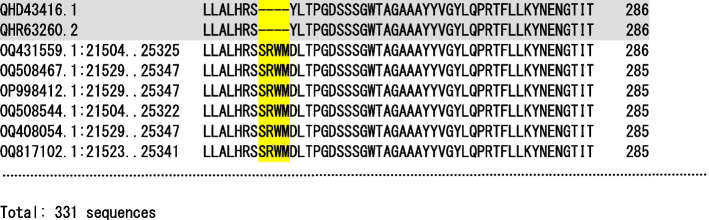
SARS-CoV-2 spike glycoprotein SRWM insert at the N-terminal domain. Header of a fragment the SARS-CoV-2 spike glycoprotein multiple sequence alignment including the reference sequences [22] and sequences identified with the S247 SRWM insert (coordinate based on reference sequence) at the N-terminal domain. The total number of sequences in the multiple alignment is 331, however, for simplicity the figure only shows a part. Sequence are identified by SARS-CoV-2 GenBank genome accession and S gene coordinates. The insert is highlighted in yellow. At the top, the reference sequences are shaded in grey. Dashes denote gaps. The numbers on the right indicate sequence position

**Fig. 5:**
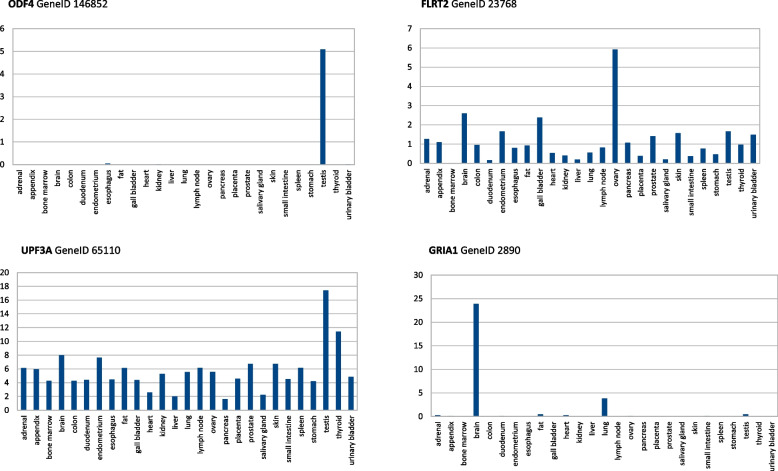
Tissue specificity of human genes matching SARS-CoV-2 S gene coding SRWM insert at the N-terminal domain. Example of human gene tissue-specificity of genes (referenced in Table 6) whose mRNA transcripts 100% match the SARS-CoV-2 S gene insert encoding the S protein N-terminal domain SRWM insert. Data were download from NCBI Human Genome Resources, assembly GRCh38.p14. Transcript expression units are normalized reads per kilobase of transcript, per million mapped reads (RPKM)

As an example, the S247 serine S, arginine R, tryptophan W, methionine M (SRWM) insert at the N-terminal domain is discussed (Fig. 4). Unlike PRRA, the acquisition of the SRWM insert was probably not associated with a known gain-of-function. Regarding the SRWM insertion, any sequencing errors could be ruled out. In the study sample, the insert has been identified in 331 SARS-CoV-2 isolates, having 15 different submitters from 15 different organizations. All isolates were from USA, but from 26 different states (collection dates November 2022–March 2030). Table 8S shows detailed information of the SRWM related virus isolates. Like the furin PRRA insertion, the SRWM coding region and its reverse complement 100% to several human mRNA transcripts. Table 6 summarizes the related genes which are also ubiquitous and highly expressed or specific of target organs or tissues of virus infection. Figure 5 shows examples of tissue-specificity of these genes.

**Table 6:** Human genes whose mRNA 100% match the SARS-CoV-2 S gene insert encoding the spike glycoprotein N-terminal domain SRWM motif

S gene insert	Ubiquitous gene	Ubiquitous and highly expressed^a^ gene	Virus target organ or tissue specific gene	Human SARS-CoV-2 target organ or tissue
Coding TCAAGATGGATG	PANX1, SLC9A8, ESPL1, ARHGAP22, ERCC6	HNRNPM, CYTH3	ODF4 PEX5 UPF3A ZBTB21 FLRT2 GRIA1	Testis Testis Testis Adrenal Ovary Brain
Reverse complement CATCCATCTTGA	LSM8		IQCE ST6GAL2 SPIRE1	Testis Thyroid Brain

^a^hight expressed, RPKM > 10 (transcript expression units are normalized reads per kilobase of transcript, per million mapped reads, RPKM)

Table 9S and Fig. 1S show details of further PRRA-like insertions in the N-terminal domain (T109, insert ILWW; T299, insert AVPY), in the ACE2 receptor binding domain (RBD) (V483, insert EVQF) and in the furin cleavage site itself (N679, insert GIAL).

The spike glycoprotein PRRA-like insertions strongly suggest a recombination between the SARS-CoV-2 genome and human mRNA transcripts within an infected human cell. In this sense, the acquisition of PRRA may not have been a single event of recombination. As well as the S gene insert encoding the PRRA motif, the S gene inserts encoding the S protein PRRA-like inserts do not appear to be of viral origin.

## Conclusions

The 12-nucleotide fragment of the SARS-CoV-2 that encoded the first identified spike glycoprotein furin polybasic motif (PRRA) 100% matches to several human mRNA transcripts. This is the basis for the hypothesis of the probable human origin of that fragment that was imprinted into the viral S gene. The hypothesis fits with the possibility that a progenitor of SARS-CoV-2 passed to humans, acquiring the PRRA during undetected human-to-human transmission.

The hypothesis is supported by:

- The knowledge that Laos and Vietnam bats host the closest known relatives to SARS-CoV-2 can infect human cells despite the absence of the furin cleavage motif [6, 7].
- The nature of human genes whose transcripts 100% match the furin S gene insert. They are ubiquitous and highly expressed genes or specific of target genes of tissues virus infection.
- Synonymous base substitution at the SARS-CoV-2 furin arginine pair CGG-CGG codon, suggesting a SARS-CoV-2 evolution to adapting the arginine codon usage.
- PRRA-like insertions at the spike glycoprotein strongly suggest that the fragments inserted in the S gene that encode them do not have a viral origin and were acquired by recombination. The PRRA acquisition may not have been a single event of recombination.

## Methods

The source of information was: (*i*) National Center for Biotechnology Information (NCBI) Virus database, SARS-CoV-2 Data Hub [23] and (*ii*) Global Initiative on Sharing Avian Influenza Data (GISAID) database [22, 24]. The reference SARS-CoV-2 S Gene and spike glycoprotein sequences were retrieved from the SARS-CoV-2 reference genomes: (*i*) Wuhan-Hu-1 isolate, GenBank: QHD43416.1 coded by MN908947.3:21563–25,384; and (*ii*) GISAID, EPI_ISL_406801 isolate, genome hCoV-19/Wuhan/WH04/2020: 21551–25,370 [14]. A pipeline of scripts in Perl for data management has been created. The rationale of this work was based on the following tasks:

### Task 1. Getting sequences

NCBI SARS-CoV-2 spike glyprotein sequences and coding regions were retrieved from the NCBI Virus database. GISAID SARS-CoV-2 spike glyprotein sequences and coding regions required data parse by executing several chained programs. Briefly:

- Download the complete genomes of the virus isolates
- To retrieve the genome regions covering the S gene (coordinates between 20,000–26,000).
- Using NCBI BLASTn [25], to identify the spike glycoprotein coding region: start and end coordinates. Query: flanking regions of the reference NCBI S gene; subject: the set of genomic regions downloaded from the GISAID database covering the S gene.
- Based on the start- and end-coordinates, to retrieve the S gene region from the downloaded GISAID genomic regions.
- To translate forward three frames of the retrieved S gene regions (coding region).
- To identify the proper translation reading frame (no ambiguous characters, no stop signals). As a result, the spike glycoprotein sequences have been obtained.
- Based on the proper reading frame, to adjust the spike glycoprotein coding regions. As a result, the S gene sequences have been obtained.

### Task 2. Similarity analysis between the SARS-CoV-2 S gene insert encoding PRRA and the human mRNA transcripts

The human transcripts database was download from NCBI Human Genome Resources (RefSeq Transcripts, GRch38, download date 05/08/2023). The Reference Sequence (RefSeq) collection provides a comprehensive, integrated, non-redundant, well-annotated set of sequences, including genomic DNA, transcripts, and proteins [12]. The downloaded version had 184,489 mRNA sequences grouped in four accession series, denoting the following: “NM_”, curated mRNA protein-coding trancripts (66,826 sequences); “NR_”, RNA non-protein-coding trascripts (20,584); “XM_”, mRNA predicted model protein-coding transcript (69,354); and “XR_”, RNA predicted model non-protein-coding transcript (27,725). The sequence analysis similarity was performed using the SARS-CoV-2 S gene 12 nucleotide insert as a query. For each SARS-CoV-2 S gene 12-nucletide insert PRRA coding, and for each human mRNA RefSeq transcript sequence a 12-nucletide window was run through the entire human mRNA sequence. The 100% match were reported.

### Task 3. Arginine codon usage bias or synonymous base substitution in the arginine pair of the SARS-CoV-2 furin site

Because the insertion of the PRRA created a novel RRAR furin cleavage site (with another R after the A in the sequence) that introduces two arginine codons CGG–CGG, using the “RRAR” query sequence that was run as four position window through the entire protein sequence the spike glycoprotein RRAR motif was identified. The protein RRAR motif position was multiplied by three to obtain the corresponding S gene codons. The cases in which the pair of the arginine codons were different from CGG-CGG were recorded.

### Task 4. To identify PRRA-like insertions in the SARS-CoV-2 spike glycoprotein sequences

NCBI Virus protein sequences were downloaded with the following filters: host human, ambiguous characters (X) 0, and sequence length 1260–1300. Using a Perl script, the downloaded protein sequences were grouped into blocks of 4000 sequences to be used thorough the EMBL Clustal Omega tool [26], which can align up to 4000 sequences or a maximum file size of 4 MB. The reference SARS-CoV-2 spke glycoprotein sequences were included in each block. Then, using another Perl script, each large multiple sequence alignment was computationally analysed. The spike glycoprotein sequences generating four or more gaps strictly conserved in all other sequences in the block were identified. Then, the coding region of the identified sequences (having the insert) and the reference S gene sequence were aligned using the HIV Sequence Database Codon Alignment v2.1.0 tool [27, 28]. The nucleotide fragment encoding the protein insert was identified.

### Task 5. Tissue-specificity of human genes related to the SARS-CoV-2 PRRA and PRRA-like insertions

The human genes under study were those whose mRNAs had made a 100% match with the S gene inserts. Tissue specificity was retrieved from the Human Protein Atlas RNA-seq normal tissues (HPA RNA-seq normal tissues. NCBI BioProject, Accession: PRJEB4337 ID: 231263). In this BioProject, based on 95 human individuals, the normal human gene expression was determined in 27 different normal human organ or tissues. Data were download from NCBI Human Genome Resources, assembly GRCh38.p14. Units of transcript expression are normalized reads per kilobase of transcript, per million mapped reads (RPKM). The results are shown in bar graphs.

### Supplementary Information

**Additional file 1: Table 1S.** NCBI human mRNA RefSeq transcripts matching the CTCCTCGGCGGG SARS-CoV-2 S gene insert.

**Additional file 2: Table 2S.** NCBI human mRNA RefSeq transcripts matching the TCCTCGGCGGGC SARS-CoV-2 S gene insert.

**Additional file 3: Table 3S.** NCBI human mRNA RefSeq transcripts matching the CCCGCCGAGGAG SARS-CoV-2 S gene insert.

**Additional file 4: Table 4S.** NCBI human mRNA RefSeq transcripts matching the GCCCGCCGAGGA SARS-CoV-2 S gene insert.

**Additional file 5: Table 5S.** NCBI Virus database SARS-CoV-2 isolates with synonymous base substitution at the arginine codons CGG-CGG.

**Additional file 6: Table 6S.** GISAID database SARS-CoV-2 isolates with synonymous base substitution at the arginine codons CGG-CGG.

**Additional file 7: Table 7S.** GISAID database SARS-CoV-2 lineages with synonymous base substitution at the arginine codons CGG-CGG.

**Additional file 8: Table 8S.** NCBI Virus database SARS-CoV-2 isolates with spike gycoprotein N-terminal domain SRWM insert.

**Additional file 9: Table 9S.** NCBI human mRNA RefSeq transcripts matching SARS-CoV-2 S gene inserts encoding S protein PRRA-like.

**Additional file 10: Figure 1S.** Tissue specificity of human genes matching SARS-CoV-2 S gene coding PRRA-like insertion at the NTD, RBD and furin site.

## Data Availability

The datasets generated and/or analysed during the current study are all available in the Tables and Figures of both the article itself and the Supplementary Information. All data are the results of sequence analyses. The sequences have been downloaded from NCBI Virus and GISAID databases. In all datasets, the sequence identifier (id) is indicated. The datasets generated in this study are not the type to be uploaded to a life science digital content repository, such as: proteomics data and/or Protein sequences; DNA and/or RNA sequences; genetic polymorphisms; linked genotype and/or phenotype data; Macromolecular structure; gene expression data; or crystallographic data.
