# Supplementary material for: Probable human origin of the SARS-CoV-2 polybasic furin cleavage motif
Source: BMC Genom Data. 2023 Nov 21;24:71. doi: 10.1186/s12863-023-01169-8 (PMC10664542; doi:10.1186/s12863-023-01169-8)
Supplement: Supplementary file 1 [file 12863_2023_1169_MOESM10_ESM.odt]

|  | B3GALT5 beta-1,3-galactosyltransferase 5 [Homo sapiens (human)] Gene ID: 10317 Chromosome, 21   │   Location, NC_000021.9 (39612940..39673137) |
| --- | --- |
| RPKM | gráfico |
|  | SCIN scinderin [Homo sapiens (human)] Gene ID: 85477 Chromosome, 7   │   Location, NC_000007.14 (12570720..12660182) |
| RPKM | gráfico |

Figure 1S

|  | WDR72 WD repeat domain 72 [Homo sapiens (human)] Gene ID: 256764 Chromosome, 15   │   Location, C_000015.10 (53513741..53762878, complement) |
| --- | --- |
| RPKM | gráfico |
|  | MAPK4 mitogen-activated protein kinase 4 [Homo sapiens (human)] Gene ID: 5596 Chromosome, 18   │   Location, NC_000018.10 (50559612..50731826) |
| RPKM | gráfico |

Figure 1S (continued)

|  | NUP214 nucleoporin 214[Homo sapiens (human)] Gene ID: 8021 Chromosome, 9   │   Location, NC_000009.12 (131125586..131234663) |
| --- | --- |
| RPKM | gráfico |
|  | CHKB choline kinase beta [Homo sapiens (human)] Gene ID: 1120 Chromosome, 22   │   Location, NC_000022.11 (50578963..50582849, complement) |
| RPKM | gráfico |

Figure 1S (continued)

|  | TMEM174 transmembrane protein 174 [Homo sapiens (human)] Gene ID: 134288 Chromosome, 5   │   Location, NC_000005.10 (73173193..73175143) |
| --- | --- |
| RPKM | gráfico |
|  | ZFHX4 zinc finger homeobox 4 [Homo sapiens (human)] Gene ID: 79776 Chromosome, 8   │   Location, NC_000008.11 (76681247..76867281) |
| RPKM | gráfico |

Figure 1S (continued)

|  | EPHA6 EPH receptor A6 [Homo sapiens (human)] Gene ID: 285220 Chromosome, 3   │   Location, NC_000003.12 (96814594..97761532) |
| --- | --- |
| RPKM | gráfico |
|  | SLAMF9 SLAM family member 9 [Homo sapiens (human)] Gene ID: 89886 Chromosome, 1   │   Location, NC_000001.11 (159951492..159983984, complement) |
| RPKM | gráfico |

Figure 1S (continued)

|  | MYH1 myosin heavy chain 1 [Homo sapiens (human)] Gene ID: 4619 Chromosome, 17   │   Location, NC_000017.11 (10492307..10518542, complement) |
| --- | --- |
| RPKM | gráfico |

Figure 1S

Figure 1S. Tissue specificity of human genes matching SARS-CoV-2 S gene coding PRRA-like insertion at the NTD, RBD and furin site

Figure 1S.  Examples of tissue specificity of human genes referenced in Tables 9S whose NCBI mRNA RefSeq transcripts 100% match the SARS-CoV-2 S gene insert encoding of the spike glycoprotein N-terminal domain (T109, insert ILWW; T299, insert AVPY), ACE2 receptor binding domain (RBD) (V483, insert EVQF) and in the furin cleavage site itself (N679, insert GIAL). Expression pattern based on the Human Protein Atlas RNA-seq normal tissues NCBI BioProject Accession: PRJEB4337 ID: 231263. Data were download from NCBI Human Genome Resources, assembly GRCh38.p14. Units of transcript expression are normalized reads per kilobase of transcript, per million mapped reads (RPKM).
