# Supplementary material for: Probable human origin of the SARS-CoV-2 polybasic furin cleavage motif
Source: BMC Genom Data. 2023 Nov 21;24:71. doi: 10.1186/s12863-023-01169-8 (PMC10664542; doi:10.1186/s12863-023-01169-8)
Supplement: Supplementary file 2 [file 12863_2023_1169_MOESM9_ESM.odt]

Table 9S. NCBI human mRNA RefSeq transcripts matching SARS-CoV-2 S gene inserts encoding S protein PRRA-like insetions

| Position | Accession | Isolate | Country | Collection_Date | Lenght | Insertion | Coding | Match |
| --- | --- | --- | --- | --- | --- | --- | --- | --- |
| T109 | UHT93004 | MN-CDC-IBX008379942786 | USA | 2021-12-08 | 1275 | ILWW-113 | ATTCTATGGTGG-339 | yes |

| #Query: ATTCTATGGTGG |  |
| --- | --- |
| #Translate: ILWW |  |
| #Query* | Transcript |
| ATTCTATGGTGG-1320 | NM_001112706.3 Homo sapiens scinderin (SCIN), transcript variant 1, mRNA |
| ATTCTATGGTGG-8710 | NM_001278650.2 Homo sapiens beta-1,3-galactosyltransferase 5 (B3GALT5), transcript variant 6, mRNA |
| ATTCTATGGTGG-8941 | NM_001356336.2 Homo sapiens beta-1,3-galactosyltransferase 5 (B3GALT5), transcript variant 7, mRNA |
| ATTCTATGGTGG-8894 | NM_001356338.2 Homo sapiens beta-1,3-galactosyltransferase 5 (B3GALT5), transcript variant 8, mRNA |
| ATTCTATGGTGG-8663 | NM_001356339.2 Homo sapiens beta-1,3-galactosyltransferase 5 (B3GALT5), transcript variant 9, mRNA |
| ATTCTATGGTGG-8849 | NM_006057.3 Homo sapiens beta-1,3-galactosyltransferase 5 (B3GALT5), transcript variant 1, mRNA |
| ATTCTATGGTGG-788 | NM_033128.3 Homo sapiens scinderin (SCIN), transcript variant 2, mRNA |
| ATTCTATGGTGG-8716 | NM_033170.3 Homo sapiens beta-1,3-galactosyltransferase 5 (B3GALT5), transcript variant 2, mRNA |
| ATTCTATGGTGG-8958 | NM_033171.3 Homo sapiens beta-1,3-galactosyltransferase 5 (B3GALT5), transcript variant 3, mRNA |
| ATTCTATGGTGG-8798 | NM_033172.3 Homo sapiens beta-1,3-galactosyltransferase 5 (B3GALT5), transcript variant 4, mRNA |
| ATTCTATGGTGG-2160 | NM_182758.4 Homo sapiens WD repeat domain 72 (WDR72), transcript variant 1, mRNA |

| #Query: CCACCATAGAAT | reverse_complement |
| --- | --- |
| #Query | Transcript |
| CCACCATAGAAT-1863 | NM_001292039.2 Homo sapiens mitogen-activated protein kinase 4 (MAPK4), transcript variant 2, mRNA |
| CCACCATAGAAT-2903 | NM_001292040.2 Homo sapiens mitogen-activated protein kinase 4 (MAPK4), transcript variant 3, mRNA |
| CCACCATAGAAT-2856 | NM_001318324.2 Homo sapiens nucleoporin 214 (NUP214), transcript variant 3, mRNA |
| CCACCATAGAAT-3279 | NM_002747.4 Homo sapiens mitogen-activated protein kinase 4 (MAPK4), transcript variant 1, mRNA |
| CCACCATAGAAT-2886 | NM_005085.4 Homo sapiens nucleoporin 214 (NUP214), transcript variant 2, mRNA |
| CCACCATAGAAT-1158 | NM_005198.5 Homo sapiens choline kinase beta (CHKB), mRNA |

| Position | Accession | Isolate | Country | Collection_Date | Lenght | Insertion | Coding | Match |
| --- | --- | --- | --- | --- | --- | --- | --- | --- |
| T299 | UHJ33999 | MN-CDC-IBX044062862928 | USA | 2021-12-01 | 1275 | AVPY-301 | GCTGTACCCTAT-903 | yes |

| #Query: ATAGGGTACAGC | reverse_complemen |
| --- | --- |
| #Query | Transcript |
| ATAGGGTACAGC-12148 | NM_001195641.2 Homo sapiens phospholipase A2 receptor 1 (PLA2R1), transcript variant 3, mRNA |
| ATAGGGTACAGC-12154 | NM_007366.5 Homo sapiens phospholipase A2 receptor 1 (PLA2R1), transcript variant 1, mRNA |

| Position | Accession | Isolate | Country | Collection_Date | Lenght | Insertion | Coding | Match |
| --- | --- | --- | --- | --- | --- | --- | --- | --- |
| V483 | UKF78016 | UT-UPHL-220202512527 | USA | 2021-12-29 | 1279 | EVQF-489 | GAGGTGCAATTT-1467 | yes |

| #Query: GAGGTGCAATTT |  |
| --- | --- |
| #Translate: EVQF |  |
| #Query* | Transcript |
| GAGGTGCAATTT-6278 | NM_003913.5 Homo sapiens pre-mRNA processing factor 4B (PRPF4B), transcript variant 1, mRNA |
| GAGGTGCAATTT-639 | NM_005937.4 Homo sapiens MLLT6, PHD finger containing (MLLT6), mRNA |
| GAGGTGCAATTT-942 | NM_153217.3 Homo sapiens transmembrane protein 174 (TMEM174), mRNA |

| #Query: AAATTGCACCTC | reverse_complement |
| --- | --- |
| #Query* | Transcript |
| AAATTGCACCTC-1672 | NM_001040402.3 Homo sapiens defective in cullin neddylation 1 domain containing 4 (DCUN1D4), transcript variant 1, mRNA |
| AAATTGCACCTC-1721 | NM_001287755.1 Homo sapiens defective in cullin neddylation 1 domain containing 4 (DCUN1D4), transcript variant 3, mRNA |
| AAATTGCACCTC-1835 | NM_001287757.2 Homo sapiens defective in cullin neddylation 1 domain containing 4 (DCUN1D4), transcript variant 4, mRNA |
| AAATTGCACCTC-11564 | NM_024721.5 Homo sapiens zinc finger homeobox 4 (ZFHX4), mRNA |

| Position* | Accession | Isolate | Country | Collection_Date | Lenght | Insertion | Coding | Match |
| --- | --- | --- | --- | --- | --- | --- | --- | --- |
| N679 | QSX93802 | Pskov-16 | Russia | 2021-01-18 | 1268 | GIAL-674 | GGGATAGCACTT-2022 | yes |

| #Query: GGGATAGCACTT |  |
| --- | --- |
| #Translate: GIAL |  |
| #Query | Transcript |
| GGGATAGCACTT-8037 | NM_001080448.3 Homo sapiens EPH receptor A6 (EPHA6), transcript variant 1, mRNA |
| GGGATAGCACTT-617 | NM_033438.4 Homo sapiens SLAM family member 9 (SLAMF9), transcript variant 1, mRNA |

| #Query: AAGTGCTATCCC | reverse_complement |
| --- | --- |
| #Query | Transcript |
| AAGTGCTATCCC-2286 | NM_005963.4 Homo sapiens myosin heavy chain 1 (MYH1), mRNA |
